# Comparison of Quetiapine and Risperidone in Treatment of Acute Psychosis: A Double-Blind, Randomized-Controlled Study

**DOI:** 10.5539/gjhs.v7n5p359

**Published:** 2015-06-10

**Authors:** S. Mohamad Moosavi, Mahshid Ahmadi, Dianoosh Mojtahedi, Jamshid Yazdani, Mani B. Monajemi

**Affiliations:** 1 Department of Psychiatry, Mazandaran University of Medical Sciences, Sari, Iran; 2 Department of Community Medicine, Mazadaran University of Medical Sciences, Sari, Iran; 3 Department of Statistics, Mazandaran University of Medical Sciences, Sari, Iran; 4 Department of Clinical Psychology, University of Tehran, Tehran, Iran

**Keywords:** quetiapine, risperidone, acute psychosis, double-blind, randomized-controlled study

## Abstract

**Objective:**

The aim of this study was to evaluate the effectiveness of Quetiapine versus Risperidone in control of acute psychotic signs and symptoms in hospitalized patients during four weeks.

**Methods:**

In this double-blind, randomized controlled study, a total of 90 patients with a confirmed diagnosis acute psychosis and were hospitalized in Zare Hospital, Sari, Iran, and they were treated with Quetiapine (mean 500 mg/day) or Risperidone (mean 5.2 mg/day), in a 4 week period. The positive and negative symptoms scale (PANSS) and Clinical Global Impression-Severity scale (CGI-s) were used to assess psychotic symptoms and severity of illness in first and the last day of the study.

**Results:**

No significant difference found between two groups in decreasing positive and negative sub-scores in the PANSS. Risperidone was superior to Quetiapine in decreasing the PANSS general psychopathology sub-scores and total score (p< 0.05). No significant difference found between two groups in decreasing CGI-s score.

## 1. Introduction

Psychosis is one of mental disorders with impairment of thought, emotional response and interactions. This group of disorders often interfere the ability of reality testing. Classic features of psychosis include hallucination and delusion (Sadock, n.d.). Suitable and in time therapeutic response, has a substantial effect on abating the symptoms of acute phase of psychosis and it may prevent the illness chronicity (Kane, Stroup, & Marder, n.d., 1547). A revolution conceived by chlorpromazine for treating psychosis in 1952 (Kane, Stroup, & Marder, n.d., 1548). Second generation antipsychotics have been available since 1990. These drugs are serotonin-dopamine receptors antagonist. The first drug of this group was Clozapine. Resperidone, Olanzapine, Quetiapine, Ziprasidone and Aripprazole were producted later (Kane, Stroup, & Marder, n.d., 1549). Second Generation Antipsychotics (SGAs) maybe more effective comparing to First Generation Antipsychotics (FGAs) in controlling positive and negatives symptoms of psychosis. Admittedly, they may have less adverse side effect and using these drugs may decrease the number of drug resistant patients. Furthermore, they may decrease the relapse rate and long-term hospitalization (Kane et al., 1549).

Regarding uprising production and using of SGAs and different results of previous studies, we decided to compare the effectiveness of two drugs of this group in acute phase of psychosis.

## 2. Materials and Methods

This was a double-blind, randomized controlled clinical trial on 90 patients with psychosis with age range of 20 to 50 years whom were admitted in Zare Hospital (Sari, Iran) from February 2013 to January 2014. Patients were randomly assigned to two groups of 45 subjects matched for age and sex. Risperidone was administrated in one group and another group was given Quetiapine. Inclusion criteria included patients with Psychotic disorders: Schizophrenia, Schizophreniform, Brief Psychotic disorder, Substance-Induced Psychotic disorder (except psychosis due to general medical condition), which have been diagnosed by psychiatrist based on DSM-IV-R with structural clinical interview.

Exclusion criteria involved patients out of defined range for age, any mood disorders (MDD with Psychotic Features, Bipolar disorders with psychotic features and Schizoaffective disorder), history of orally used antipsychotic drugs 6 weeks before evaluation and mentioning long acting antipsychotics this period defined as 8 weeks, prominent neurological and/or physical disorders based on physical and neurological examination during hospitalization period and laboratory tests (CBC, FBS, BUN, Creatinine, ALT, AST, Alkaline Phosphatase, UA and also ECG), Consuming other psychotic drug even in PRN situation, Having Electro Convulsive therapy (ECT) based on attending physician order and prescribing any drugs except Resperidone and Quetiapine during hospitalization. Regarding type of the illness and its acute phase, written consent forms were useless. Instead, this process for the study explained for the parents or legal guardian of the patients and all of them gave their consent. This study was conducted in accordance with the declaration of Helsinki & respectable clinical practice according to international conference on harmonization guidelines.

G-Power software was used and considering potency of 80, sample size was 90. Regarding to probability of exclusion for any reason and due to unpredicted problems during the study, 117 patients had been chosen for study. The patients were divided in two groups using random numbers nature and with using Excel software and Randbetween Function. Clinical interview for all patients conducted by a psychiatrist and also CGI scale (Rockville, 1976) was done in order to assess the severity of the illness. Positive and negative symptoms scale (PANSS) to specify the severity of negative and positive and general psychiatric symptoms. These scales include 7 negative symptoms, 7 positive symptoms and 14 general psychopathologic symptoms. Each symptom scored between 0-7 based on severity. This scale is being used widely in researches regarding Antipsychotic drugs. Ghamari and colleagues confirmed validity and reliability of this Scale in Iranian population pool (Ghamari Givi, Molavi, & Heshmati, 2010).

Each group of patients, were under Resperidone or Quetiapine treatment. In this study, first drug (Resperidone) was chosen from Poursina Pharmaceutical Company and the second (Quetiapine) was produced by Jam Co. Initial dosage of neuroleptic drugs in common situation based on researches and textbooks can be 1mg for Resperidone and for Quetiapine 50 mg Per-day (Marder, Hurford, & Kammen, n.d., 3236). The initial dosage was 2 mg for Resperidone and 100 mg for Quetiapine group due the acute situation of the patients in this study. Maximum daily dosage was 8 mg for Resperidone and 800 mg for Quetiapine (Marder et al., n.d., 3236). At the end of week 4, mean dosages of drug for each patient were measured. Based on statistical survey prior to this study, the average duration of hospitalization period was 27 days, so and we measured the dosage of drugs after week 4.

At the end of the study, PANSS and CGI scales assessed participants again. Patients were not notified about the drug they were taking and in order to eliminate any kind of bias, in assessment and scoring the scales; research associate didn’t have any information about drug and its dosage.

Data were analyzed by SPSS software version 19. In order to analyze data, for quantitative variables such as age and PANSS, descriptive statistics method such as Mean ± standard deviation (SD) was used and for qualitative variables distribution frequency chart was used. In order to compare the effectiveness of the drugs we used Analyze of Variance with repetitive measurement of Chi-square and one-way Analyze of Variance with Bonferoni post-test. If groups were not the same in terms of cofounding variables, Generalized Estimated Equations (GEE) method was used. P value less than 0.05 were defined as significant in this study.

## 3. Results

117 patients were included in this study. 8 patients discharged before 4 weeks, so they were excluded from our study. 6 patients needed ECT according to comments of attending physician; they were excluded from the study too. 13 patients have been under PRN. Finally, study was conducted on 90 patients.

Patients were included 15 women and 75 men. 8 women were in Resperidone group and 7 were in Quetiapine group. 37 in first group and 38 in second group were female. The Mean ± SD age of patients whom underwent Risperidone was 34.3±10.4 versus 35.5±11.2 in Quetiapine group. In this relation, there was no significant difference between the ages of study arms (p=0.422). 37(41.1%) patients in Risperidone group and 38 (42.2%) in Quetiapine group were male while 8 (8.8%) in Risperidone group and 7 (7.77%) in Quetiapine group were female. There was no significant change in studied patients gender (p=0.777) (Table 1).

**Table 1:** Age & Sex Distribution in Resperidone and Quetiapine Group

Characteristics		Treatment		P-Value
		Risperidone	Quetiapine	
Age	Mean	34.3	35.5	0.422
	SD	10.4	11.2	
Sex	Male	37	38	0.777
	Female	8	7	

In this Study, mean dosage for Resperidone was 5.6 mg and mean dosage for Quetiapine was 498 mg. 100 mg chlorpromazine is equivalent to 2 mg Resperidone and 75 mg Quetiapine. Hence, 280 mg for Resperidone with equivalent dose of Chlorpromazine and 664 mg for Quetiapine was specified. Abovementioned means we used higher dose of Resperidone in this study.

Two groups had no statistically meaningful difference in relation to CGI-s scores from start point to the end of this study (p<0.001) but effectiveness of both drugs in reducing CGI-s was meaningful. In PANSS sub-scores (General/Positive/Negative), there were no meaningful difference at baseline. At the end of week 4, no statistically difference in reducing positive (p=0.892) and negative (p=0.286) symptoms observed between Risperidone and Quetiapine groups whereas differences between two groups were significant regarding general symptoms (P=0.001) (as seen in Table 2). It means Resperidone was more effective than Quetiapine in reducing general symptom scores of PANSS in acute phase of psychosis. Furthermore, the difference between groups in total scores between two groups was statistically meaningful (P=0.04) and this confirms superior effectiveness of Resperidone over Quetiapine in treating acute phase of psychosis.

**Table 2:** Comparing PANSS AND CGI-S, Baseline and Week 4

Treatment		Baseline		Diff. After 3 weeks	

		Mean	P-value	Mean	P-value
CGI-s	*Risperidone*	4.78±0.42	0.241	2.42±0.62	0.001
	*Quetiapine*	4.87±0.41		2.56±0.79	
Positive symptoms.	*Risperidone*	24.38±2.66	0.926	10.51±2.34	0.892
	*Quetiapine*	24.33±1.81		10.58±2.30	
Negative symptoms.	*Risperidone*	18.02±3.12	0.579	3.56±1.39	0.286
	*Quetiapine*	18.38±2.93		3.87±1.35	
General symptoms.	*Risperidone*	49.27±4	0.190	18.13±2.23	0.001
	*Quetiapine*	48.18±3.82		15.8±2.71	
Total	*Risperidone*	91.67±6.07	0.638	32.2±4.07	0.040
	*Quetiapine*	91.04±6.41		30.2±4.87	

## 4. Discussion

In this double blind randomized controlled study, we examined the effect of Resperidone versus Quetiapine in management of acute phase of psychosis. This study revealed that both of these drugs could reduce the severity of acute psychosis significantly. Resperidone reduced PANSS in general symptoms scores and total scores more than Quetiapine in acute psychosis. Although differences in reducing total, positive and negative scores were not significant and both drugs had the same therapeutic effect. There was no difference in reducing positive and negative symptoms in acute psychosis between Resperidone and Quetiapine. These results were similar to results of the study of Potkin and Colleagues (Potkin et al., 2006). That study was conducted on schizophrenic patients who were in acute phase of the disorder and the therapeutic period was 6 weeks. This may explain that effectiveness of these two drugs in reducing positive and negative symptoms in different kind of psychosis with short therapeutic period is similar. The result of our study is concordant to result of the study of Yan Li and colleagues (Y. Li, H. Li, Liu, Yan, Yue, & Qian, 2012). In their study, in 6 weeks period, no significant difference was noticed in relation of reducing psychotic symptoms with 750 mg of Quetiapine and 4 mg of Resperidone on a daily dosage. Due to higher dose of Quetiapine in Yan’s study, we probably can conclude that effectiveness of both drugs may be different in each patient and it is not always necessary to equate dosage with 100 mg chlorpromazine.

8 weeks assessment conducted by Y-hua and colleagues in schizophrenic patients didn’t show difference the effectiveness of these two drugs (Ye-hua, Jun-yan, & Ying-chun, 2012), and the result is similar to the result of present study. Despite our study, in that research all of the patients were women and this can lead us to the conclusion that both drugs are effective in both gender. Furthermore, in relation to superior effectiveness of Resperidone in reducing general symptoms comparing to Quetiapine, which was mentioned in current study, result is as same as the results of other studies (Potkin et al., 2006; Johnsen & Jorgensen, 2008; Komossa, Rummel-Kluge, Schwarz, Schmid, Hunger, Kissling, & Leucht, 2011). Although, for understanding causes of similarity of Resperidone and Quetiapine effects in reducing positive and negative symptoms and differences in general scores, further investigations may be required. In Komossa and Colleagues’ review study in 2011 was shown that Quetiapine was less effective than Resperidone and Olanzapine in controlling the symptoms of psychosis (Johnsen & Jorgensen, 2008). In present study, effectiveness of Resperidone and Quetiapine in reducing acute psychotic symptoms was equal and the difference in results of two studies maybe due to difference in research’s tool, age of the patients and genetic background. In another study, which was again conducted by Komossa and colleagues, effectiveness of Resperidone was compared to other second-generation antipsychotics (SGA) and the conclusion was that Resperidone is more effective than Quetiapine in reducing PANSS (Komossa et al., 2011). In our study, the result is the same except in general scores. Although, this will draw a question that why and how Resperidone and Quetiapine can reduce positive and negative symptoms but they have different manner in general scores. This question may need further pharmacological and pathophysiological researches. In 8 weeks study conducted by Zhong with daily dose of 525 mg Quetiapine and 5.2mg Resperidone, the group treated by Resperidone showed more decreased positive symptoms (Zhong, Sweitzer, Hamer, & Lieberman, 2006). In current study, mean dose of Quetiapine was less than mean dose of Resperidone and this may explain the superior effectiveness of Resperidone. It means that, although each patient may be treated with different dose of drug but mean dose for most patients are being affected by occupancy of most dopaminergic receptors and partially serotoninergic receptors. In a study conducted by Johnson, decrease in PANSS for Quetiapine was more and faster than other drugs (Johnsen, Kroken, ToreWentzel-Larsen, & Jørgensen, 2010). It seems that, one of the differences of Johnson’s study with current study is difference of PANSS score in patients by initiation point of the study. In Johnson’s study PANSS score was 74 and in our study, the score was 91.5 by the time we started the study. Furthermore, duration of treatment and follow-up of the patients in Johnson’s study was 2 years, which means that that study covered both acute and residual phase of the disorder, but in our study, we only covered acute phase. It may be understood that Quetiapine may be more effective in long period, although, more researches may be needed regarding this assumption.

Regarding limitation of this study 4 weeks period of the study and number of samples may be mentioned. It is advised that in future researches, it is better to study larger samples in longer period.
